# Discovery of new PSII inhibitors: systematic investigation for herbicidal activity screening, properties, and mode of action

**DOI:** 10.1002/ps.70071

**Published:** 2025-07-17

**Authors:** Mirjalol Umurzokov, Danhee Kim, Jin‐Seog Kim, Kwang Min Cho, Jung Sup Choi, Kee Woong Park, Hee Nam Lim

**Affiliations:** ^1^ Daeseungbiofarm Co., Ltd. Daejeon Republic of Korea; ^2^ Chemical Research Team New Agrochemical Research Institute Farmhannong Nonsan‐si Republic of Korea; ^3^ Eco‐Friendly New Materials Research Center Korea Research Institute of Chemical Technology Daejeon Republic of Korea; ^4^ Department of Chemistry Yeungnam University Gyeongsan Republic of Korea

**Keywords:** triazine derivatives, PSII inhibition, GR₅₀ values, nonselective, contact herbicide

## Abstract

**BACKGROUND:**

The research focused on developing effective photosystem II (PSII) inhibiting triazine‐derived herbicidal compounds from the internal chemical library. Initially, the compounds were screened for their herbicidal activity using *Spirodela polyrhiza* (SPIPO) assays. Subsequently, compounds with stronger activity were synthesized on a gram scale and further investigated for their phytotoxicity, mobility, and mode of action.

**RESULTS:**

In the SPIPO screenings, two compounds, **K‐31706** and **K‐31487**, showed superior efficacy at concentrations exceeding 0.4 μm, surpassing the commonly used PSII inhibition herbicide, atrazine. Both compounds showed promising performance, especially in pre‐emergence applications under greenhouse conditions, with the application rate causing 50% biomass reduction below 100 g a.i. ha^−1^. However, both compounds exhibited strong phytotoxicity on major monocot and dicot crops, particularly when applied pre‐emergence. Absorption studies revealed that **K‐31706** and **K‐31487** have strong activity in both soil‐only and overtop applications, with root‐based absorption occurring in monocots like *Digitaria ciliaris*. **K‐31487** was effective across various application methods on *Abutilon theophrasti*, indicating dual root and leaf absorption in dicots. Nevertheless, leaf excision assays indicated that both compounds act as contact herbicides rather than systemic ones. Chlorophyll fluorescence induction (OJIP) curve and molecular docking analyses confirmed PSII inhibition, which is comparable with simazine and diuron, with suppressed electron transport via interaction with Q_B_ site amino acids of D1 protein.

**CONCLUSION:**

Overall, **K‐31706** and **K‐31487** are nonselective PSII inhibitors that block the electron transport in PSII, positioning them as potentially useful active ingredients for further investigation. © 2025 The Author(s). *Pest Management Science* published by John Wiley & Sons Ltd on behalf of Society of Chemical Industry.

## Graphical abstract

Two nonselective contact photosystem II inhibitors were discovered and investigated through various biological evaluations, including *Spirodela polyrhiza* and whole‐plant bioassays, and studies on their mobility and mode of action.

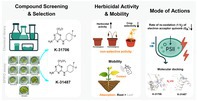

## 1 INTRODUCTION

The demand for innovative weed‐control agents has intensified because of the rapid evolution of herbicide resistance among weed species worldwide.^1^ Resistance to existing herbicides poses a significant threat to agricultural productivity, because it limits the effectiveness of traditional chemical solutions in managing weeds that compete with crops for essential resources such as light, water, and nutrients.^2^ To address these challenges, developing novel herbicidal compounds with improved efficacy and selectivity has become an increasingly significant priority in modern agricultural science.

Triazine derivatives, a class of nitrogen‐containing heterocyclic compounds, have been extensively used as herbicides for decades, primarily because of their ability to inhibit photosystem II (PSII) in target plants.^3^ Compounds such as atrazine and simazine, prominent members of the triazine family, have a crucial role in controlling various broadleaf and grass weeds in crops like corn and sorghum.^4^, ^5^ The herbicidal activity of these compounds stems from their ability to bind to the D1 protein in the PSII complex, disrupting electron flow during photosynthesis and resulting in the accumulation of reactive oxygen species that harm cellular components.^6^, ^7^ This mode of action emphasizes their effectiveness, but also reveals the risk of resistance development in weed species, as evidenced by widespread resistance in biotypes such as *Amaranthus retroflexus* and *Chenopodium album*.^8^, ^9^

The widespread use of triazine herbicides has raised considerable concerns about resistance and environmental contamination. Resistance to PSII inhibitors has been documented in various weed species around the world, while the persistence of triazine compounds in the environment has resulted in groundwater contamination and other ecological issues.^1^, ^10^ These challenges have prompted research aimed at designing new triazine derivatives with better efficacy, lower environmental persistence, and increased crop selectivity.^11^ Recent advances in synthetic chemistry have made it possible to explore structural modifications to the triazine core, leading to the discovery of compounds with unique modes of action or selective phytotoxicity against specific weed species.^12^, ^13^

Crop selectivity, a vital component of herbicide development, ensures that the herbicide effectively targets weeds while minimizing harm to crop species.^14^ Selectivity is influenced by the molecular characteristics of the herbicide and the physiological traits of the crop, which affect the compound's absorption, translocation, and metabolism.^15^ Developing herbicides with improved selectivity not only enhances their efficacy in integrated weed management strategies, but also reduces nontarget activity, herbicide residues, and environmental impact.^7^ This is particularly crucial in the context of sustainable agriculture, where the focus is on balancing weed control with environmental sensitivity stewardship.

Understanding the absorption, translocation, and mode of action of herbicides is essential for optimizing their efficacy and addressing resistance issues.^16^ Absorption typically occurs through plant surfaces, including leaves and roots, and is influenced by the physicochemical properties of the herbicide, environmental conditions, and the structure of the plant cuticle.^17^ After absorption, systemic herbicides must translocate through the xylem or phloem to reach their target sites in photosynthetically active or meristematic tissues.^6^, ^18^, ^19^ Effective translocation is especially crucial for perennial or deeply rooted weed species, where the herbicide's distribution within the plant affects its bioavailability and potency.^20^

Resistance to herbicides with common modes of action, such as PSII inhibitors or acetolactate synthase inhibitors, has led researchers to explore novel compounds with unique biochemical targets or altered patterns of absorption and translocation.^21^, ^22^ These new modes of action present an opportunity to overcome resistance and broaden the scope of effective weed‐control options.^16^ By identifying compounds that utilize different physiological processes, researchers aim to develop herbicides that are both effective against resistant weed species and environmentally sustainable.

In this study, the herbicidal activity was screened using 2,6‐diamino‐1,3,5‐triazine derivatives stored in an internal chemical library; the *Spirodela polyrhiza* (SPIPO) bioassay developed by Kim and Kim^23^ was used for assessing herbicidal activity. The most potent compounds were synthesized on a gram scale, and detailed bioassays were conducted to assess their herbicidal efficacy, mode of action, and crop selectivity. In addition, we investigated the absorption and translocation characteristics of the selected compounds to understand their mobility within plant tissues and their interactions with biochemical targets.

## 2 MATERIALS AND METHODS

### 2.1 Herbicidal activity screenings in the SPIPO bioassay

The selected internal library compounds were preliminary tested for their herbicidal activity using the SPIPO herbicidal activity screening method.^23^, ^24^ The SPIPO was grown in plastic tanks with 5 L of Steinberg medium at pH 6.9, cultured at constant temperature (25 ± 1 °C) under a 14 h photoperiod, and 20–30 μmol m^−2^ s^−1^ of fluorescent light. Each compound was dissolved in a suitable solvent such as dimethylformamide (DMF) or dimethyl sulfoxide and diluted to the desired concentrations in distilled water with Tween‐80. Fronds with four similarly sized green leaves were selected and placed in a transparent plastic cup (90 mL) containing 30 mL of medium with 0.4, 4, and 40 μm concentrations of compounds. Phytotoxic activity (visual investigation %) was assessed 5 days after treatment (DAT). Atrazine, a commercialized herbicide, was used as a positive control. Compounds that showed the highest herbicidal activity were selected and used in further detailed studies.

### 2.2 Synthesis of selected triazine derivatives

#### 2.2.1 Materials and reagents

Solvents, including methanol, dichloromethane, and deionized water were directly used after purchase from TCI Chemicals (Tokyo, Japan) and Duksan Chemicals (Daejeon, Republic of Korea). Thin‐layer chromatography analysis was run on silica gel plates. Most of the spots were visualized by exposure to ultraviolet light (254 nm). Nuclear magnetic resonance (NMR) and high‐resolution mass spectrometry (HRMS) spectra confirmed structure of the synthesized triazine derivatives; they were recorded using VNMR 600 MHz (600 MHz for ^1^H and 150 MHz for ^13^C), and Vanquish UHPLC High‐Resolution Mass System with ion trap (orbitrap) mass analyzer (electrospray ionization mode) at Core Research Support Center for Natural Products and Medical Materials at Yeungnam University.

#### 2.2.2 Synthesis procedure

**Scheme 1:**
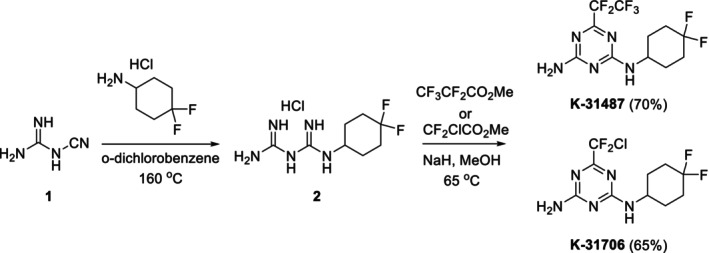
Synthesis of the triazine derivatives

The synthetic route to access compounds **K‐31487** and **K‐31706** is described in Scheme 1. The addition reaction of 4,4‐diflurocyclohexylamine into 1‐cyanoguanidine (**1**) in heated *o*‐dichlorobenzene afforded **2** as the common intermediate for triazine synthesis. Construction of the triazine core was enabled by the reactions between **2** and methyl 2,2,3,3,3‐pentafluoropropanate or methyl 2‐chloro‐2,2‐difluoroacetate in the presence of sodium methoxide in methanol, giving **K‐31487** and **K‐31706** in 70% and 65% yields, respectively. The structure of each synthesized triazine compound was confirmed by ^1^H and ^13^C NMR spectroscopy. The molecular weight and structure were further verified by HRMS.

### 2.3 Detailed herbicidal activity assays

The herbicidal activity of **K‐31706** and **K‐31487** in pre‐ and post‐emergence applications was assessed on four monocots and four dicot weed species (Table 1) under greenhouse conditions. **K‐31706** and **K‐31487** were dissolved in 100% DMF and diluted in tap water containing 0.5% DMF and 0.05% Tween‐80. The commercial herbicide simazine, along with tap water containing 0.5% DMF and 0.05% Tween‐80, served as positive and negative controls, respectively, in all experiments. Application doses of the compounds were adjusted to 31.25, 62.5, 125, 250, 500, and 1000 g a.i. ha^−1^, with three replicates for each herbicide treatment and the untreated plants. The aboveground parts of treated plants were harvested at 30 DAT for pre‐emergence applications and at 10 DAT for post‐emergence applications. The harvested samples were kept in an oven at 40 °C for 3 days. The dry weight of each weed species was measured and converted to herbicidal activity (%) by comparison with the dry weight of untreated (negative) control plants. The following nonlinear regression dose–response (Eqn (1)), developed by Streibig,^25^ was used to analyze the data. The log‐logistic curve was plotted using OriginPro 8.1.^26^

(1) $$y=d+\frac{a-d}{\left[1+{\left(\frac{x}{c}\right)}^{b}\right]}$$

where *y* = dry weight, *d* = the maximum value, *a* = the minimum value, *x* = the dose of chemicals, *c* = GR_50_ (the rate causing 50% biomass reduction), and *b* = response line slope around the rate of GR_50_.

**Table 1:** List of weed species used in herbicidal activity assays

Type	Scientific name	Common name
Grass weeds	*Panicum dichotomiflorum*	Autumn millet
	*Lolium perenne*	Perennial ryegrass
	*Echinochloa crus‐galli*	Barnyard grass
	*Digitaria ciliaris*	Crabgrass
Broadleaf weeds	*Amaranthus retroflexus*	Pigweed
	*Solanum nigrum*	Black Nightshade
	*Abutilon theophrasti*	Velvetleaf
	*Xanthium strumarium*	Common cocklebur

#### 2.3.1 Pre‐emergence application

Weed species listed in Table 1 were seeded in one rectangular plastic pot (350 cm^2^) filled with commercial paddy soil, which was divided into two columns and four rows. One day after seeding, selected test compounds (**K‐31706** and **K‐31487**), the positive and negative controls, were applied at a volume of 25 mL per pot. Treated plants were grown in a greenhouse (30/20 ± 5 °C, light/dark = 12/12 h).^27^ The herbicidal activity was evaluated at 30 DAT.

#### 2.3.2 Post‐emergence application

Weed species, given in Table 1, were seeded in one rectangular plastic pot (350 cm^2^) containing commercial horticulture soil, which was divided into two columns and four rows. Fifteen days after seeding (two‐ to three‐leaf stage), the seedlings were treated with **K‐31706** and **K‐31487**, positive and negative controls, at the volume of 25 mL pot^−1^. The herbicidal activity was assessed at 10 DAT.

### 2.4 Crop selectivity screening

Selected crop species, including rice, wheat, soybean, and Chinese cabbage, were seeded separately in pots (50 cm^2^), which were filled with paddy soil for pre‐emergence application and commercial horticulture soil for post‐emergence application. One day after seeding, **K‐31706** and **K‐31487**, positive and negative controls, were applied at a volume of 7 mL pot^−1^ at 31.25, 62.5, 125, 250, 500, and 1000 g a.i. ha^−1^ to the soil surface (pre‐emergence application). For post‐emergence application, treatments were sprayed onto the foliage when seedlings were in the three‐ to five‐leaf stage. After spraying, the treated plants were kept in the greenhouse (30/20 ± 5°C, light/dark = 12/12 h). Phytotoxic activity was assessed at 21 DAT (pre‐emergence) and 7 DAT (post‐emergence) following the method described for the herbicidal activity assays.

### 2.5 Herbicide mobility

#### 2.5.1 Absorption

Seeds of *Digitaria ciliaris* and *Abutilon theophrasti* were sown and grown to the two‐ to three‐leaf stage in plastic pots (50 cm^2^) filled with commercial horticultural soil. **K‐31706** and **K‐31487** were dissolved in 100% DMF and subsequently diluted in tap water containing 0.5% DMF and 0.05% Tween‐80. The commercial herbicide simazine, combined with tap water that also contained 0.5% DMF and 0.05% Tween‐80, served as the positive and negative controls, respectively. The herbicide solutions were applied in three methods: foliar‐only, soil‐only, and overtop sprayed (foliar + soil). The application doses of the chemicals were adjusted to 31.25, 62.5, 125, 250, 500, and 1000 g a.i. ha^−1^. For the soil‐only applications, the herbicides were applied using a micropipette directly onto the soil surface, ensuring there was no contact with the weeds. For foliar‐only and overtop spray applications, **K‐31706**, **K‐31487**, and simazine were applied using a spray gun. In the case of the foliar‐only application, the soil surface was covered with dry sand to a depth of 1 cm just before spraying. Once the treatment was completed, the sand was removed from the pots. Each herbicide treatment and the untreated plants included three replicates.

The aboveground parts of the surviving plants were harvested at 10 DAT and placed in an oven at 40 °C for 3 days. The dry weights of *D. ciliaris* and *A. theophrasti* were measured separately and converted to herbicidal activity (%) in comparison with the dry weight of untreated (negative) control plants. The nonlinear regression dose–response (Eqn (1)) was used to analyze the data. The log‐logistic curve was drawn using the OriginPro 8.1 program.^26^

#### 2.5.2 Translocation

**Figure 1:**
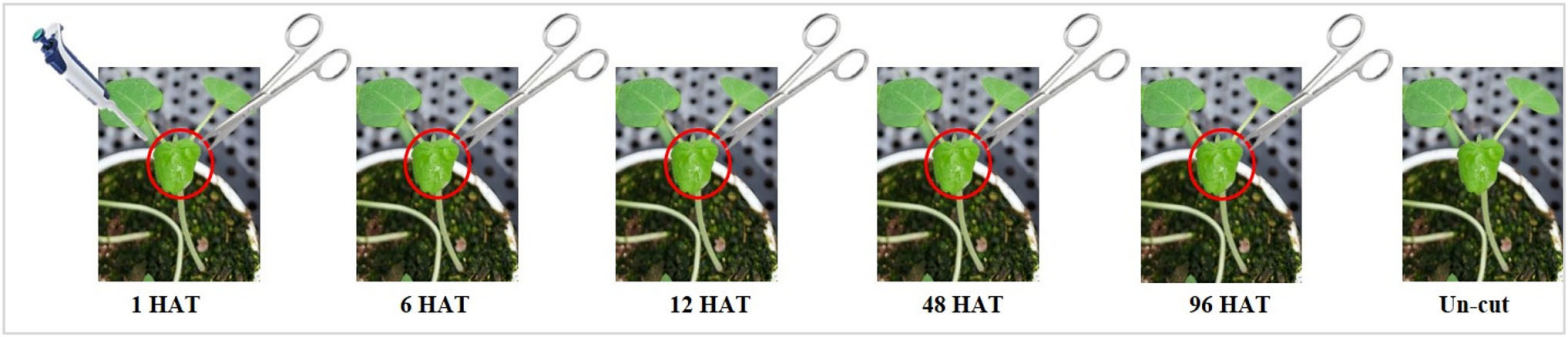
The experimental procedure and sample harvesting time.

The leaf excision bioassay, as described by Koo,^28^ evaluates the absorption and translocation speed of a foliar‐applied, systemic herbicide. In this method, the herbicide is applied to a fully developed leaf, which is later excised at various time intervals. If a lethal dose is absorbed and translocated to essential tissues, the plant will die even after leaf removal; if removed too early, the plant may survive. By analyzing plant responses over time, this method enables comparison of absorption and translocation rates among herbicides with similar modes of action. In this study, *D. ciliaris* (monocot) and *A. theophrasti* (dicot) were grown under greenhouse conditions (30/25 ± 5 °C, light/dark = 14/10 h). At the three‐leaf stage of plant growth, 50 μL per plant of **K‐31706**, **K‐31487**, and simazine were applied at 20 μg a.i. plant^−1^ using a micropipette to the middle of the second leaf on the seedlings. The treated leaves were then excised at 1, 6, 12, 48, and 96 h after treatment (HAT) (Fig. 1). Each herbicide treatment, the uncut control (the treated leaf not excised), and the untreated control were replicated three times. Herbicidal activity (0, no activity; 100, complete death) was visually evaluated at 7 DAT.

### 2.6 Mode of action

#### 2.6.1 Chlorophyll fluorescence OJIP transient

The effects of the tested compounds on the chlorophyll fluorescence OJIP transient of cucumber leaf tissues were assessed and compared with those of the commercial herbicides, simazine and diuron. In the OJIP curve, “O” stands for “origin” (minimal fluorescence), “P” for “peak” (maximum fluorescence), and J and I for inflection points between the O and P levels. The treatments were applied at concentrations of 0.1, 1.0, and 10 μm. OJIP transients were measured at 6, 24, 48, and 96 h post‐treatment using a Fluorpen FP100/D (Photon System Instruments, Drásov, Czech Republic). The first measurable point of the fluorescence transient was recorded at *t*_0_ = 50 μs and was designated *F*_0_, representing the initial fluorescence intensity. Relative variable fluorescence at the J step (*V*_j_) in the chlorophyll fluorescence intensity curve was quantified using Eqn (2). Rapid chlorophyll fluorescence transients obtained under these conditions were subsequently analyzed to evaluate the rate of reoxidation (1 – *V*_j_) of the primary acceptor quinone (Q_A_). The parameter of 1 – *V*_j_ is used to detect and assess the power of inhibitors displacing the secondary acceptor quinone (Q_B_) from its binding pocket in the D1 protein.^29^, ^30^, ^31^ A lower value of (1 – *V*_j_) indicates a lower reoxidation rate, meaning the electron transfer from Q_A_ to Q_B_ is slower or less efficient.

(2) $$V_{j}=\left(\left(F_{j}–F_{0}\right)/\left(F_{M}–F_{0}\right)\right)$$

Where *F*_j_ is the fluorescence intensity measured at 2 ms after illumination begins, *F*_0_ is the initial fluorescence intensity measured at 50 μs, and *F*_M_ is the maximum fluorescence intensity recorded during the transient.

#### 2.6.2 Molecular docking analyses

The crystal structure (2.70 Å) of D1 protein (chain A, PDB: 5XNL) was obtained from the Protein Data Bank (PDB) database and used as the receptor.^32^ Three‐dimensional structures of **K‐31706**, **K‐31487** and simazine were built in Open Babel,^33^ and were used as ligands. AutoDock Tools version 1.5.7 was used to prepare ligands and receptor, and molecular docking was carried out with AutoDock 4.2.6^34^ to predict binding modes. For each compound, 50 genetic algorithm runs were performed with a population size of 300. The docking results were evaluated based on the internal scoring functions of AutoDock Tools. The docked conformations were ranked based on the binding energy of the ligand, and the conformation with the lowest binding energy was chosen as the best binding pose.^35^

### 2.7 Statistical analyses

The statistical analyses for the entire study were done using one‐way analysis of variance in OriginPro 8.^26^ Fisher's least significant difference (LSD) test with a significance level of *P* < 0.05 was used to compare the means.

## 3 RESULTS

### 3.1 Herbicidal activity screenings in SPIPO bioassay

**Figure 2:**
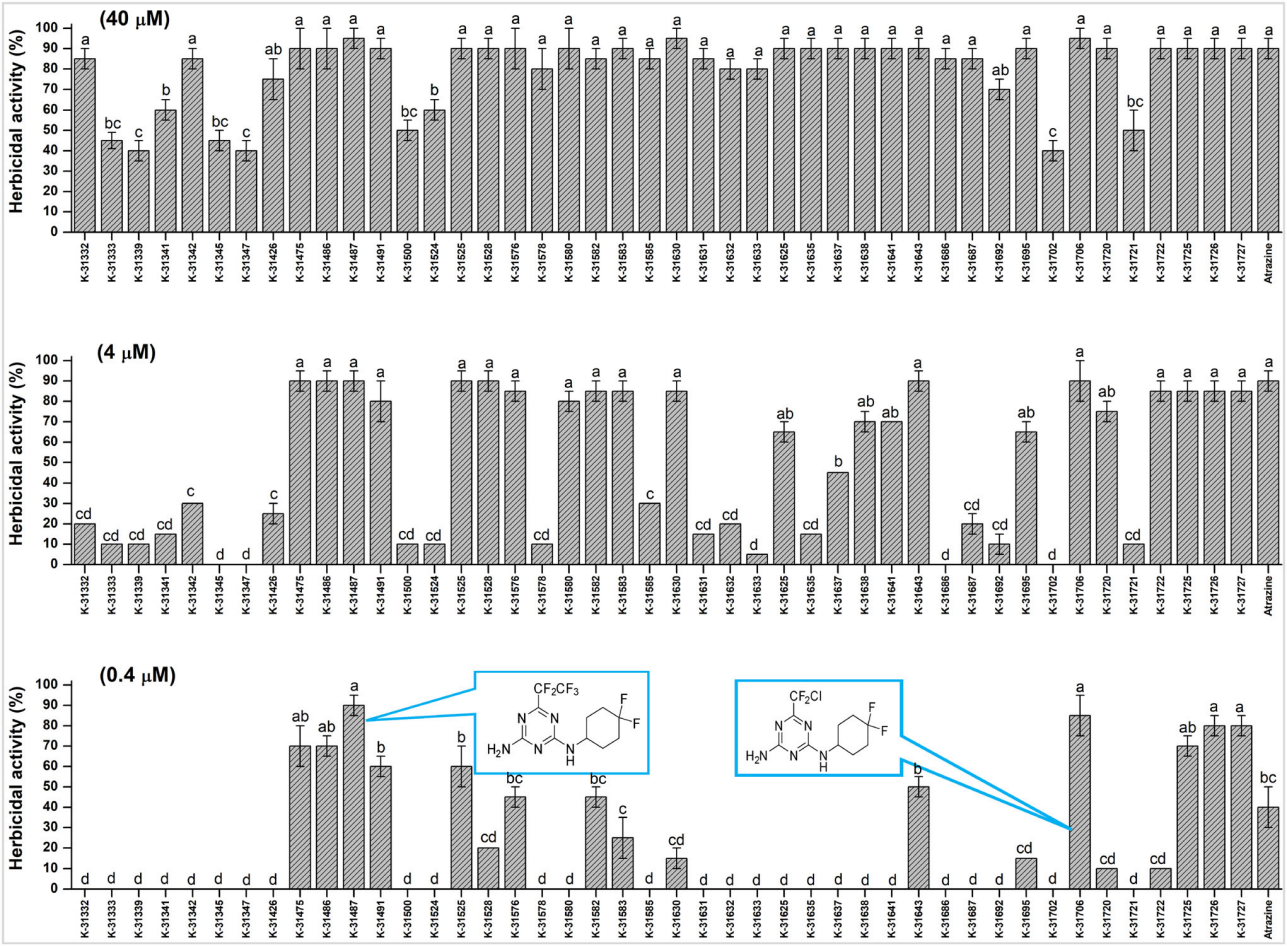
Herbicidal activity screening of triazine‐derived chemical compounds at three different concentrations using the *Spirodela polyrhiza* bioassay at 5 days after treatment. Vertical bars indicate the standard error of the mean, and different letters denote significant differences in Fisher's LSD test (*P* < 0.05).

In total, 224 triazine‐derived compounds from the chemical library were assessed in the SPIPO bioassay at a concentration of 40 μm (data not shown). Of these, 44 compounds exhibited substantial herbicidal effects, with >40% phytotoxicity. These compounds were further screened for herbicidal activity at reduced concentrations of 4 and 0.4 μm. Among the 44 compounds, **K‐31706** and **K‐31487** consistently demonstrated high herbicidal activity (>90% inhibition), surpassing the commercial standard, atrazine (Fig. 2). Thus, **K‐31706** and **K‐31487** were chosen for further investigations, which included detailed assessments of herbicidal activity, crop selectivity assays, herbicide mobility, and the modes of action of these compounds.

### 3.2 Detailed herbicidal activity assays

#### 3.2.1 Pre‐emergence application

In the pre‐emergence application, increasing doses of the tested compounds led to a progressive reduction in dry weight across all monocot and dicot weed species. Across all species, both **K‐31706** and **K‐31487** showed dose‐dependent reductions in plant dry weight, with the most substantial effects occurring at higher doses (>100 g a.i. ha^−1^). With the exception of *L. perenne*, the application rate causing 50% biomass reduction (GR₅₀) for all weed species treated with **K‐31706**, **K‐31487**, and simazine in pre‐emergence applications were below 100 g a.i. ha^−1^ (Table 2). Among them, *A. retroflexus* was the most sensitive species, displaying GR₅₀ values below 30 g a.i. ha^−1^.

**Table 2:** GR_50_ values of monocot and dicot weed species for **K‐31706**, **K‐31487**, and simazine in the pre‐emergence application

Treatments	GR₅₀ (g a.i. ha^−1^)							
	Grass weeds				Broadleaf weeds			
	*Panicum dichotomiflorum*	*Lolium perenne*	*Echinochloa crus‐galli*	*Digitaria ciliaris*	*Amaranthus retroflexus*	*Solanum nigrum*	*Abutilon theophrasti*	*Xanthium strumarium*
**K‐31706**	51.8a ± 5.4	166.7a ± 10.5	52.8a ± 5.8	50.8a ± 5.7	25.9ab ± 0.6	48.6a ± 5.1	50.5b ± 7.2	59.8b ± 6.4
**K‐31487**	49.5a ± 5.7	174.7a ± 10.6	51.6a ± 5.5	49.4a ± 5.9	22.3a ± 0.7	24.2b ± 2.8	39.6b ± 5.8	80.2a ± 7.9
Simazine	47.7a ± 5.1	149.3b ± 8.9	50.8a ± 6.1	50.2a ± 6.8	29.3b ± 0.6	47.4a ± 4.9	71.6a ± 7.5	74.5a ± 7.7

*Note*: Numbers following ± are the standard error of the mean and different letters in each column indicate significant differences in Fisher's LSD test (*P* < 0.05).

Abbreviations: GR_50_, rate causing 50% biomass reduction.

#### 3.2.2 Post‐emergence application

Similar to the pre‐emergence application, post‐emergence treatment with increasing doses of **K‐31706**, **K‐31487**, and simazine resulted in a progressive reduction in dry weight across all grass and broadleaf weed species. Among the tested herbicides, **K‐31487** exhibited greater growth inhibition than **K‐31706** and the commercial herbicide simazine at all concentrations and across all weed species. The GR₅₀ values of weed species were generally higher in post‐emergence applications than in pre‐emergence applications. However, **K‐31487** consistently exhibited the lowest GR₅₀ values among all tested species (Table 3). With the exception of *L. perenne*, the GR₅₀ values of other weed species for **K‐31706** and **K‐31487** were below 100 g a.i. ha^−1^. Specifically, *L. perenne* exhibited GR₅₀ values of 163.6 g a.i. ha^−1^ for **K‐31706** and 110 g a.i. ha^−1^ for **K‐31487**. Overall, both **K‐31706** and **K‐31487** showed slightly greater herbicidal activity against broadleaf weed species compared to grass weed species in the post‐emergence applications.

**Table 3:** GR_50_ values of monocot and dicot weed species for **K‐31706**, **K‐31487**, and simazine in the post‐emergence application

Treatments	GR₅₀ (g a.i. ha^−1^)							
	Grass weeds				Broadleaf weeds			
	*Panicum dichotomiflorum*	*Lolium perenne*	*Echinochloa crus‐galli*	*Digitaria ciliaris*	*Amaranthus retroflexus*	*Solanum nigrum*	*Abutilon theophrasti*	*Xanthium strumarium*
**K‐31706**	62.6ab ± 8.7	163.6b ± 15.6	61.6b ± 5.7	110.4b ± 9.7	38.2a ± 1.5	45.7a ± 1.0	58.7b ± 7.2	98.8b ± 7.0
**K‐31487**	44.2b ± 5.9	110.0c ± 10.0	53.7b ± 4.8	90.9b ± 7.9	35.3a ± 1.4	42.6a ± 1.0	27.8c ± 6.8	74.8b ± 5.3
Simazine	88.7a ± 9.7	222.5a ± 24.4	113.5a ± 10.2	192.1a ± 16.3	43.5a ± 1.4	47.9a ± 1.0	213.2a ± 28.9	200.2a ± 13.8

*Note*: Numbers following ± are the standard error of the mean and different letters in each column indicate significant differences in Fisher's LSD test (*P* < 0.05).

Abbreviations: GR_50_, rate causing 50% biomass reduction.

### 3.3 Crop selectivity screening

#### 3.3.1 Monocot crop species

In pre‐emergence applications, **K‐31487** showed the highest herbicidal potency in rice, with a GR₅₀ value of 30.4 g a.i. ha^−1^, followed closely by **K‐31706** at 43.9 g a.i. ha^−1^ (Table 4). By contrast, simazine required a substantially higher dose of 367.1 g a.i. ha^−1^ to achieve GR₅₀. In post‐emergence applications, the GR₅₀ values of rice were 57.9, 40.3, and 185.6 g a.i. ha^−1^ for **K‐31706**, **K‐31487**, and simazine, respectively.

**Table 4:** GR_50_ values of *Oryza sativa* and *Triticum aestivum* for **K‐31706**, **K‐31487**, and simazine in pre‐ and post‐emergence applications

Crop species	Treatments	GR₅₀ (g a.i. ha^−1^)	
		Pre‐emergence application	Post‐emergence application
*O. sativa*	**K‐31706**	43.9b (4.4)	57.9b (4.7)
	**K‐31487**	30.4b (3.7)	40.3b (4.3)
	Simazine	367.1a (43.1)	185.6a (14.4)
*T. aestivum*	**K‐31706**	34.9b (3.8)	32.2b (4.7)
	**K‐31487**	25.3b (4.3)	25.4b (4.8)
	Simazine	672.2a (88.3)	373.1a (57.9)

*Note*: Numbers in parentheses are the standard error of the mean and different letters in each column indicate significant differences in Fisher's LSD test (*P* < 0.05).

Abbreviations: GR_50_, rate causing 50% biomass reduction.

For wheat, the GR₅₀ values in pre‐emergence applications were 34.9 g a.i. ha^−1^ for **K‐31706** and 25.3 g a.i. ha^−1^ for **K‐31487**. In post‐emergence treatments, the GR₅₀ values of what were 32.2 g a.i. ha^−1^ and 25.4 g a.i. ha^−1^ for **K‐31706** and **K‐31487**, respectively (Table 4).

#### 3.3.2 Dicot crop species

In pre‐emergence applications, **K‐31487** demonstrated the strongest phytotoxic activity in soybean, with a GR₅₀ value of 36.5 g a.i. ha^−1^, followed by **K‐31706** at 43.3 g a.i. ha^−1^ (Table 5). Simazine required an application rate exceeding 1000 g a.i. ha^−1^ to reach GR₅₀ in soybean dry weight reduction. In post‐emergence applications, the GR₅₀ values for soybean were 108.0, 78.1, and 530.6 g a.i. ha^−1^ for **K‐31706**, **K‐31487**, and simazine, respectively.

**Table 5:** GR_50_ values of *Glycine max* and *Brassica pekinensis* for **K‐31706**, **K‐31487**, and simazine in pre‐ and post‐emergence applications

Crop species	Treatments	GR₅₀ (g a.i. ha^−1^)	
		Pre‐emergence application	Post‐emergence application
*G. max*	**K‐31706**	43.3c (5.7)	108.0b (11.0)
	**K‐31487**	36.5c (4.8)	78.1c (8.9)
	Simazine	1,486.9a ± (62.8)	530.6a (66.0)
*B. pekinensis*	**K‐31706**	26.0c (1.9)	40.7b (1.6)
	**K‐31487**	22.7c (1.9)	39.3b (1.5)
	Simazine	369.9b (19.6)	111.4a (3.9)

*Note*: Numbers in parentheses are the standard error of the mean and different letters in each column indicate significant differences in Fisher's LSD test (*P* < 0.05).

Abbreviations: GR_50_, rate causing 50% biomass reduction.

For Chinese cabbage, the GR₅₀ values in pre‐emergence applications were 26.0 g a.i. ha^−1^ for **K‐31706** and 22.7 g a.i. ha^−1^ for **K‐31487**. In post‐emergence treatments, the GR₅₀ values were 40.7 and 39.3 g a.i. ha^−1^ for **K‐31706** and **K‐31487**, respectively (Table 5).

### 3.4 Herbicide mobility

#### 3.4.1 Absorption

**Figure 3:**
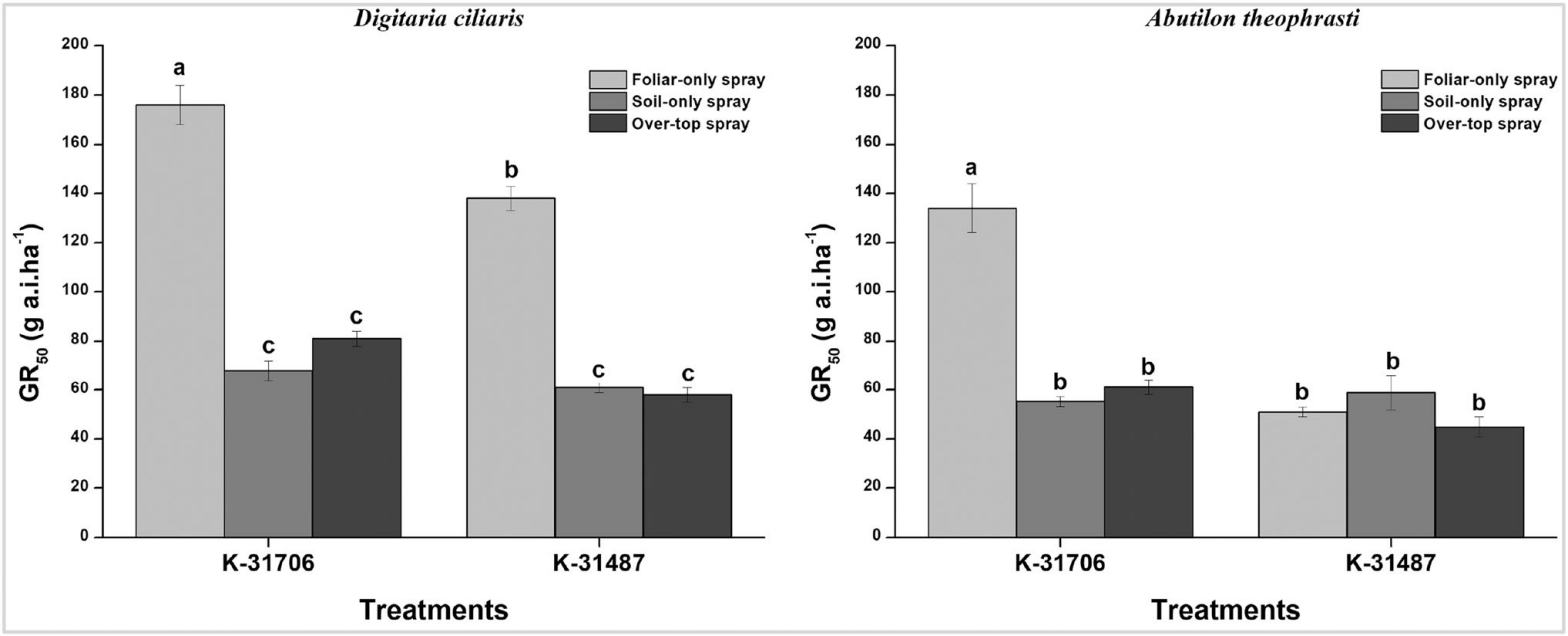
GR_50_ value of *Digitaria ciliaris* and *Abutilon theophrasti* for **K‐31706** and **K‐31487** in different application methods. Vertical bars indicate the standard error of the mean and different letters indicate significant differences in Fisher's LSD test (*P* < 0.05).

**K‐31706** and **K‐31487** effectively controlled *D. ciliaris*, achieving GR_50_ values below 100 g a.i ha^−1^ in both soil‐only and overtop spray applications (Fig. 3). The GR_50_ values for *D. ciliaris* were 170 g a.i ha^−1^ for **K‐31706** and 140 g a.i ha^−1^ for **K‐31487** in foliar‐only applications. **K‐31706** showed superior activity in soil‐only sprays, whereas **K‐31487** demonstrated greater efficacy in overtop spray applications. This indicates that absorption primarily occurs through the hypocotyl or root in monocot weed species. The GR_50_ value for *A. theophrasti* was approximately 50 g a.i ha^−1^ for **K‐31487** across all three application methods (Fig. 3). Thus, the efficacy of **K‐31487** remained consistently high regardless of application position, outpacing **K‐31706** in all scenarios. This implies that absorption of **K‐31487** may involve leaf, hypocotyl and root in broadleaf weed species, whereas **K‐31706** appears to be absorbed primarily through the hypocotyl and root, rather than the leaf, in both monocot and dicot weed species.

#### 3.4.2 Translocation

In the leaf excision bioassay, both compounds exhibited similar herbicidal activity against *D. ciliaris* and *A. theophrasti* (Table 6). The growth reduction observed in both weed species, by the test compounds, was 10%, identical to that of the un‐excised control (visual rating 10%). No significant differences were found between the untreated control and the test compounds for either species. Simazine showed no herbicidal activity when leaf samples were cut 12 and 48 HAT on *D. ciliaris* and *A. theophrasti*, respectively. Overall, **K‐31706**, **K‐31487**, and simazine did not exhibit a consistent response over time. It was concluded that although both compounds are absorbed by leaf, hypocotyl or root, they are not translocated through them. In other words, these compounds function as contact herbicides rather than systemic ones.

**Table 6:** Visual rating of the *Digitaria ciliaris* and *Abutilon theophrasti* treated with **K‐31706**, **K‐31487**, and simazine in leaf excision bioassay

Herbicides	Weed species	Visual rating (0–100)					
		Excision time after treatment					
		1 h	6 h	12 h	48 h	96 h	Uncut
Untreated control	*A. theophrasti*	0a ± 0	0a ±0	0a ± 0	0b ± 0	0b ± 0	**0b ± 0**
	*D. ciliaris*	0a ± 0	0a ±0	0a ± 0	0b ± 0	0b ± 0	**0b ± 0**
**K‐31706**	*A. theophrasti*	10a ± 0	10ab ± 0	10a ± 0	10ab ± 0	10ab ± 0	**10ab ± 0**
	*D. ciliaris*	10a ± 0	10a ± 0	10a ± 0	10ab ± 0	10ab ± 0	**10ab ± 0**
**K‐31487**	*A. theophrasti*	10a ± 0	10a ± 0	10a ± 0	10ab ± 0	10ab ± 0	**10ab ± 0**
	*D. ciliaris*	10a ± 0	10a ± 0	10a ± 0	20a ± 0	20a ± 0	**20a ± 0**
Simazine	*A. theophrasti*	0a ± 0	0a ± 0	0a ± 0	10ab ± 0	10ab ± 0	**10ab ± 0**
	*D. ciliaris*	0a ± 0	0a ± 0	10a ± 0	10ab ± 0	10ab ± 0	**10ab ± 0**

*Note*: Numbers following ± are standard error of the mean and different letters in each column indicate significant differences in Fisher's LSD test (P < 0.05).

### 3.5 Mode of actions

#### 3.5.1 Chlorophyll fluorescence OJIP transient

**Figure 4:**
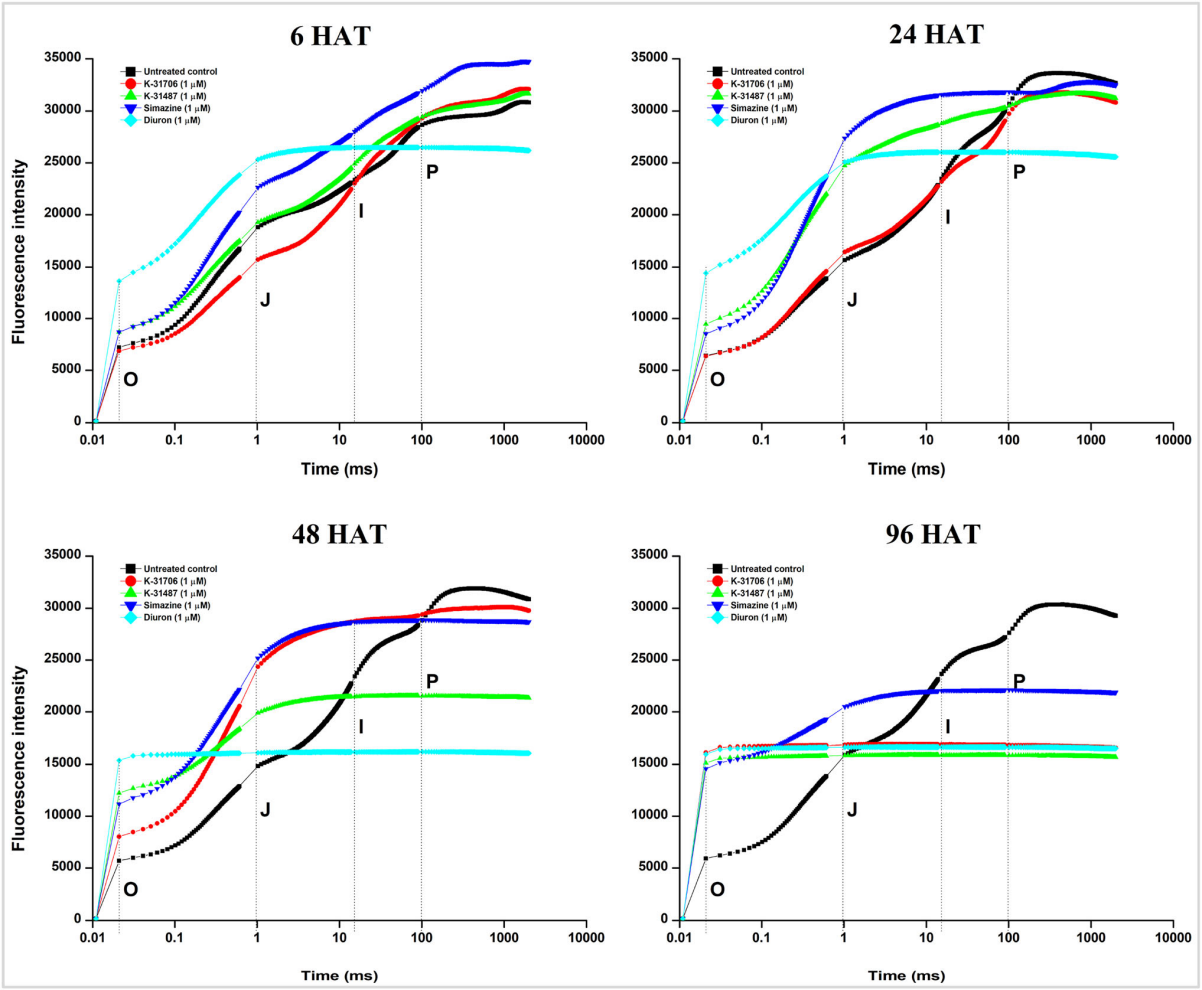
Responses of chlorophyll fluorescence OJIP transient to treatments with **K‐31706**, **K‐31487**, simazine, and diuron at a 1.0 μm concentration measured at 6, 24, 48, and 96 h after treatment.

The fluorescence intensity was measured to confirm if the mode of action of the test compounds is linked to the inhibition of electron transport within PSII. At early time points (6 HAT), no significant differences in fluorescence intensity in all levels (O, J, I, and P) were observed between the untreated control and **K‐31706**‐, **K‐31487**‐, and simazine‐treated samples at a concentration of 1.0 μm (Fig. 4). Although the diuron, which is a strong PSII inhibitor, caused alterations in the intensity curve at the J step (Fig. 4), by 48 HAT, the fluorescence intensity curves for all treated samples were suppressed, maintaining a flat pattern with reduced progression through the J, I, and P steps compared with the untreated control. This reduced response suggested sustained inhibition of electron transport caused by the treatments. By 96 HAT, the trend observed at 48 HAT persisted, and the intensity was further deceased, with treated samples continuing to show significantly lower fluorescence intensity in the J, I, and P steps relative to the untreated control.

Changes in the rate of reoxidation (1 − *V*_j_) of the primary electron acceptor quinone (Q_A_^−^) following treatment with various concentrations of **K‐31706**, **K‐31487**, simazine, and diuron at different time points are shown in Table 7. These values indicate the level of electron transfer from Q_A_ to Q_B_ acceptors in D1 protein of the PSII.

**Table 7:** The rate of reoxidation (1 − *V*_j_) of the electron acceptor quinone (Q_A_^−^) in response to treatments with **K‐31706**, **K‐31487**, simazine, and diuron at different concentrations measured at 6, 24, 48, and 96 h after treatment (HAT)

Time	Rate of reoxidation (1 − *V*_j_)												
	Untreated control	K‐31706			K‐31487			Simazine			Diuron		
		0.1 μm	1 μm	10 μm	0.1 μm	1 μm	10 μm	0.1 μm	1 μm	10 μm	0.1 μm	1 μm	10 μm
6 HAT	0.64a	0.63a	0.63a	0.12c	0.61a	0.53a	0.07c	0.64a	0.44ab	0.11c	0.35b	0.02c	0.00c
24 HAT	0.62a	0.56a	0.37b	0.04	0.39b	0.25b	0.00	0.55a	0.14bc	0.07c	0.04c	0.00c	0.00c
48 HAT	0.61a	0.44b	0.17c	0.00c	0.23bc	0.08c	0.00c	0.27bc	0.11c	0.00c	0.00c	0.00c	0.00c
96 HAT	0.54a	0.07c	0.00c	0.00c	0.04c	0.00c	0.00c	0.24b	0.10c	0.00c	0.00c	0.00c	0.00c

*Note*: Values are the mean of three replications and different letters in each row indicate significant differences in Fisher's LSD test (*P* < 0.05).

At 6 HAT, minimal differences were observed between the untreated control and low concentrations (0.1 and 1 μm) of all treatments, suggesting that effective electron transfer was still occurring at this early stage. However, at a concentration of 10 μm, all treatments had in significantly reduced 1 – *V*_j_ values (Table 7). For instance, 1 – *V*_j_ values for **K‐31706** and **K‐31487** decreased to 0.12 and 0.07, respectively, whereas diuron resulted in complete inhibition (0.00), indicating early blockage of electron transfer within the D1 protein. By 24 and 48 HAT, the decrease in the rate of reoxidation became more evident, particularly at concentrations of 1 and 10 μm. Finally, **K‐31706** and **K‐31487** completely blocked the electron transfer at 1 and 10 μm by 96 HAT. Although some measurable activity remained at the 0.1 μm concentration (0.07 for **K‐31706** and 0.04 for **K‐31487**), these values were not statistically different from those observed at 1 and 10 μm.

Overall, the analysis of chlorophyll fluorescence characteristics supports the hypothesis that **K‐31706** and **K‐31487** function as PSII inhibitors. They disrupt the electron transfer within the D1 protein complex of PSII, in a manner comparable with known herbicides such as simazine and diuron.

#### 3.5.2 Molecular docking analyses

**Figure 5:**
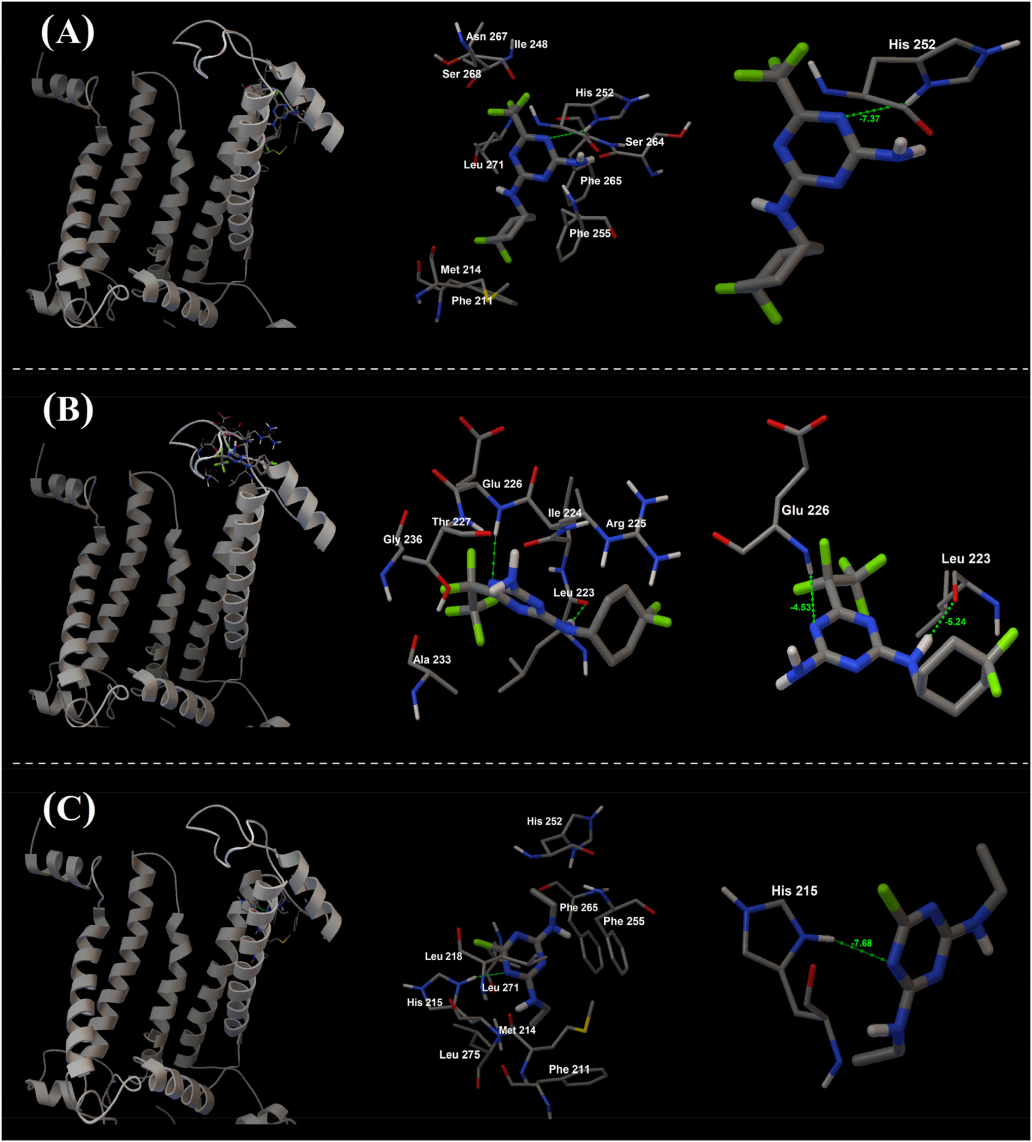
Molecular docking analyses illustrating the interaction between selected compounds and the Q_B_ binding site of the D1 protein (chain A, PDB: 5XNL). A–C, Binding geometry of **K‐31706** (A), **K‐31487** (B), and simazine (C). For each compound, the images on the left show their positioning in the D1 protein sequence, followed by a zoomed‐in view of the region highlighting the amino acid residues in close proximity. Images on the right side of each panel detail the hydrogen bonds between the ligand compounds and amino acid residues, with the hydrogen bonds and their calculated binding energies (kcal mol^−1^) in green.

The binding conformations and amino acid interactions of **K‐31706**, **K‐31487**, and simazine, as determined by molecular docking analyses, are summarized in Fig. 5. Notably, a distinct dipole–dipole interaction (hydrogen bond) was observed between the nitrogen atom in the triazine ring of **K‐31706** and the ND1 atom of His252 within the Q_B_ binding site of the D1 protein (PDB ID: 5XNL). The significantly lower binding energy of −7.37 kcal mol^−1^ (Fig. 5(A)) suggests a higher binding affinity, indicating that **K‐31706** is likely to bind strongly to the amino acid residue. In addition, several other nonpolar interactions were formed with Phe211, Met214, Ile248, Phe255, Phe265, Ser264, Asn267, Ser268, and Leu271. By contrast, **K‐31487** exhibited two key hydrogen bonds: one between its nitrogen atom in the triazine ring and amino acid residue of Glu226 (binding energy −4.53 kcal mol^−1^), and another between its amino group and amino acid residue of Leu223 (binding energy −5.24 kcal mol^−1^) within the Q_B_ binding site (Fig. 5(B)). Simazine was oriented within the Q_B_ binding site near His215, forming a polar hydrogen bond through the nitrogen atom of its triazine ring (Fig. 5(C)). It showed a hydrogen bond with relatively lower binding energy (−7.68 kcal mol^−1^), indicating higher affinity. Additional hydrophobic interactions with Phe211, Met214, His252, Phe255, and Phe265 further stabilized its binding.

## 4 DISCUSSION

Although we do not detail structure–activity relationships in this paper, several key factors regarding the structure of **K‐31706** and **K‐31487** are worth considering. Fluorinated alkyl groups (e.g., —CF_2_Cl and —CF_2_CF_3_) at the C4 position and a difluorocyclohexyl group on the C2 amino group of 1,3,5‐triazine framework appear to increase herbicidal efficacy, likely because of their improved molecular binding affinity and lipophilicity.^36^ These structural features corroborate existing literature on the role of electron‐withdrawing substituents in increasing herbicidal efficacy.^22^, ^37^, ^38^

In this study, a number of compounds showed broad‐spectrum efficacy and high activity at increased (40 and 4 μm) concentrations in the SPIPO bioassay. Subsequent screenings led to the identification of only two compounds (**K‐31706** and **K‐31487**) as potent candidates. **K‐31706** and **K‐31487** showed consistent and robust herbicidal efficacy at even the lowest (0.4 μm) concentrations, either exceeding or equaling the performance of the commercial standard, atrazine.

Following the selection of **K‐31487** and **K‐31706** as lead compounds, their herbicidal efficacy was further assessed in various weed species through pot experiments conducted under greenhouse conditions. Both compounds demonstrated dose‐dependent reductions in weed biomass, consistent with previous herbicidal efficacy studies that report a typical sigmoidal dose–response relationship for weed suppression.^39^, ^40^ The pre‐emergence application of both **K‐31706** and **K‐31487** was particularly effective, achieving substantial biomass reduction at relatively low GR₅₀ values (generally below 50 g a.i. ha^−1^ for most species), which are lower than the GR₅₀ values reported for many commercially available herbicides used in monocot weed control.^41^, ^42^, ^43^

Both monocot and dicot weed species were highly sensitive to **K‐31706** and **K‐31487** in both pre‐emergence and post‐emergence applications, indicating that **K‐31706** and **K‐31487** are broad‐spectrum herbicidal active compounds. Notably, a negative correlation was observed between the efficacy of the test compounds and weed maturity, because the herbicidal activity declined with advancing leaf stages.^44^ At early growth stages (two or three leaves), both compounds effectively suppressed *D. ciliaris* and *A. theophrasti*, whereas at later growth stages (six or seven leaves), the weeds became more tolerant. In crop selectivity assays, **K‐31706** and **K‐31487** showed significant phytotoxic effects on rice, wheat, soybean, and Chinese cabbage, particularly in pre‐emergence applications, suggesting the need for caution when applying these compounds near sensitive crops.

**Scheme 2:**
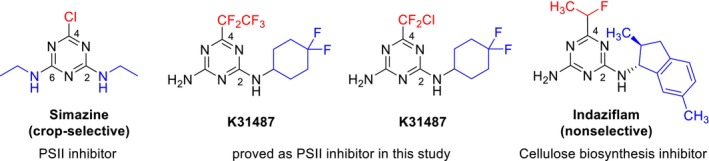
Structural analysis of **K‐31706** and **K‐31487**

The mode of action and crop selectivity of the two newly discovered compounds are analyzed in terms of their structural features, using two representative herbicides sharing triazine scaffolds. The different substitution patterns between simazine and these two new compounds are likely the key factors for crop selectivity. As shown in Scheme 2, the two amino groups at the C2 and C6 positions of simazine are substituted by primary alkyl groups, such as two ethyl groups. By contrast, only the amino group at C2 position of **K‐31487** and **K‐31706** is substituted by secondary cycloalkyl groups. Furthermore, these compounds have fluoroalkyl substituents at the C4 position instead of chloride, which distinctly differs from known crop‐selective PSII inhibitors such as atrazine, cyanazine, propazine, simazine, and tebuthylazine. Looking at the substitution patterns, we identified structural similarities between the two new compounds and indaziflam, a potent nonselective inhibitor of cellulose biosynthesis.^45^ We presume that the fluorinated alkyl group at the C4 position and *N*‐cycloalkyl group at C2 position contribute to the nonselective nature in some tested crops, while still delivering superior herbicidal activities. It is noteworthy that, despite the similar substitution patterns to indaziflam, the mode of action found in the two compounds is PSII inhibition. Conducting extensive studies on *N*‐alkylation at the C6 position and/or *N*‐cycloalkylation at the C2 position, with the variations in lipophilicity and electronic properties, could enhance crop selectivity while maintaining high herbicidal efficacy.

Subsequently, the herbicidal mobility of two novel compounds was characterized through absorption and translocation studies,^28^ and multiple bioassays were conducted to determine their mode of action.^46^ A different herbicidal efficacy was observed based on application methods. It was found that both compounds are primarily root‐mobile in grassweeds (*D. ciliaris*). For broadleaf weeds (*A. theophrasti*), **K‐31487** showed equal efficacy across application methods, suggesting dual absorption through both leaf and root. This dual mobility is critical for effective weed control, where herbicides translocate via both vascular systems can enhances overall efficacy.^47^ However, the relatively limited phloem translocation observed in **K‐31706** and **K‐31487** treatments suggests that their primary action is contact‐based rather than systemic, aligning with prior studies of limited phloem‐mobility herbicides.^48^, ^49^

The OJIP fluorescence curve is indicative of PSII electron transport efficiency. In this study, both compounds demonstrated characteristics of PSII inhibitors, as evidenced by the reduced Q_A_^−^ reoxidation and the altered chlorophyll fluorescence OJIP curves.^31^ By 96 HAT, the suppression of the OJIP fluorescence induction curve further confirmed the inhibition of PSII electron transport. This was observed as an increased intensity in the O and J transients, along with a consistent decline or stabilization in fluorescence intensity in the J, I, and P steps. An increase in the O and J transients indicates accumulation of the reduced primary acceptor Q_A_^−^, whereas the decrease in the J, I, and P transients reflects the impaired reduction of electron acceptors through PSII complex.^50^, ^51^

All the herbicides tested in the OJIP fluorescence assay exhibited concentration‐ and time‐dependent inhibition of Q_A_^−^ reoxidation (1 − *V*_j_), highlighting their significant impact on PSII electron transport. At early exposure (6 HAT), low concentrations (0.1–1 μm) of **K‐31706** and **K‐31487** caused minimal disruption of Q_A_^−^ reoxidation, comparable with commercialized PSII‐inhibiting herbicides such as simazine and diuron.^29^, ^52^ However, as time progressed, the inhibitory effects intensified, resulting in almost complete suppression of Q_A_^−^ reoxidation by 96 HAT, even at a concentration of 0.1 μm. These results are consistent with the known mechanism of PSII‐inhibiting herbicides, which bind to the D1 protein at the Q_B_ site, blocking electron transport and interrupting the reoxidation of Q_A_^−^.^53^, ^54^, ^55^, ^56^

In this study, molecular docking analyses further revealed that both **K‐31706** and **K‐31487** compounds interacted within the Q_B_ binding site of the D1 protein, a known target site for PSII‐inhibiting herbicides.^29^, ^53^, ^56^ **K‐31706** formed a single strong hydrogen bond between the nitrogen atom of its triazine ring and amino acid residue of His 252. By contrast, **K‐31487** exhibited two distinct hydrogen bonds with amino acid residues Glu226 and Leu223. Both compounds formed hydrogen bonds with relatively lower binding energy (> −4.5 kcal mol^−1^), indicating strong binding affinity and stable, effective interactions within the D1 protein.^57^, ^58^ Moreover, distinct binding behaviors of two triazine‐derived compounds revealed notable variation within the same chemical class in their ability to interact with the Q_B_ biding site. This variation might be related to the different substitutions of the fluorinated alkyl group at the C4 position.

In conclusion, compounds **K‐31706** and **K‐31487** showed nonselective broad‐spectrum contact‐based PSII‐inhibitory activity in both pre‐ and post‐emergence applications, making them promising candidates for further optimization. To develop a crop‐selective herbicide, further structural modifications are underway in our laboratory.

## CONFLICT OF INTEREST

The authors have declared that no conflict of interest exist.

## Supporting information


**Data S1:** Supporting Information.

## Data Availability

The data that supports the findings of this study are available in the supplementary material of this article.
