# Supplementary material for: Discovery of new PSII inhibitors: systematic investigation for herbicidal activity screening, properties, and mode of action
Source: Pest Manag Sci. 2025 Jul 17;81(10):7219–31. doi: 10.1002/ps.70071 (PMC12441759; doi:10.1002/ps.70071)

***Supporting Information***

Discovery of new PSII inhibitors: systematic investigation for herbicidal activity screening, properties, and mode of action

**(Running title: *Discovery of new PS II inhibitors*)**

Mirjalol Umurzokov^1+^, Danhee Kim^2+^, Jin-Seog Kim^1^, Kwang Min Cho^1^, Jung Sup Choi^3^, Kee Woong Park^1^ and Hee Nam Lim^4*^

^1^Daeseungbiofarm Co., Ltd., Daejeon 34127, Republic of Korea

^2^Chemical Research Team, New Agrochemical Research Institute, Farmhannong, Nonsan-si, Chungcheongnam-do, 33010, Korea

^3^Eco-friendly New Materials Research Center, Korea Research Institute of Chemical Technology, Daejeon 34114, Korea

^4^Department of Chemistry, Yeungnam University, 280 Daehak-ro, Gyeongsan, Gyeongbuk 35841, Republic of Korea

^+^ These authors contributed equally to this work.

^*^Corresponding author:

Hee Nam Lim: [heenam@yu.ac.kr](mailto:heenam@yu.ac.kr)

1. Materials and Methods S2
2. Experimental Procedures and Characterization Data S2-3
3. ^1^H-NMR, ^13^C-NMR, HRMS S4-6

# Materials and Methods

Common solvents and reagents were directly used after purchase from TCI chemicals without further purification. Thin layer chromatography (TLC) analysis was run on silica gel plates. Most of spots were visualized by exposure to ultraviolet (UV) light (254 nm). Some spots that were invisible to ultraviolet (UV) used PMA stain solution. NMR and HRMS spectra were recorded using Bruker VNMR 600 MHz (600 MHz for ^1^H, and 150 MHz for ^13^C) and Vanquish UHPLC High Resolution Mass System with ion trap (orbitrap) mass analyzer [Ionization mode: ESI] at Core Research Support Center for Natural Products and Medical Materials at Yeungnam University.

High-resolution mass spectra were reported for the molecular ion [M+Na]^+^ or [M+H]^+^. Chemical shifts for proton NMR spectra are reported in parts per million (ppm) relative to the singlet at 7.26 ppm for chloroform-*d*. Chemical shifts for carbon NMR spectra are reported in 77.16 ppm with the center line of triplet for chloroform-*d*. Data for ^1^H NMR were presented as following: chemical shifts (δ, ppm), multiplicity (br = broad, s = singlet, d = doublet, t = triplet, q = quartet, m = multiplet), coupling constant (Hz), and integration..

#
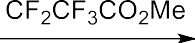
Experimental Procedures and Characterization Data


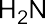

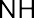

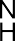

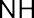

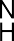

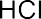

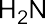

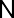

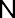

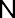

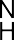

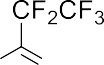


**
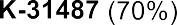
**


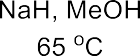
To a stirred solution of **2** (1.02 g, 4.0 mmol) in MeOH (10 mL) was added NaH (0.29 g, 4.4 mmol, 60% dispersed in mineral oil) at room temperature and the mixture was stirred for 30 min. Then, methyl pentafluoropropionate (0.54 mL, 4.2 mmol) was added to the reaction mixture. After stirring at 65 ^o^C for 18 h, the reaction was cooled to room temperature, quenched with sat. aqeous NH4Cl solution, and extracted with ethyl acetate (30 X 3 mL). The organic solution was dried over MgSO4, filtered, concentrated, and subjected to silica gel column chromatography (Hex:EtOAc = 5:1 to 1:1) to afford **K-31487** (0.97 g, 70%) as white solid.

**N2-(4,4-difluorocyclohexyl)-6-(perfluoroethyl)-1,3,5-triazine-2,4-diamine (K-31487)**; mixture of conformers (1.7:1); ^1^H NMR (600 MHz, CDCl3) δ 6.16-5.92 (m, 2.7H), 5.40-5.30 (m, 4.4H), 5.16 (m,

1H), 4.00-3.93 (m, 2.7H), 2.13-1.59 (m, 21.6H). ^13^C NMR (150 MHz, CDCl3) δ 167.2, 166.8, 165.6,

165.2, 165.2 (t, *J*C,F = 21.7 Hz), 164.8 (t, *J*C,F = 24.8 Hz), 122.5 (t, *J*C,F = 239.3 Hz), 122.4 (t, *J*C,F = 240.1

Hz), 118.7 (qt, *J*C,F = 286.1, 35.3 Hz), 118.6 (qt, *J*C,F = 285.8, 32.8 Hz), 109.1 (t, *J*C,F = 255.3 Hz), 108.8

(t, *J*C,F = 256.0 Hz), 47.9, 47.6, 32.3 (t, *J*C,F = 24.8 Hz, 2C), 28.5 (d, *J*C,F = 9.0 Hz), 28.3 (d, *J*C,F = 9.3 Hz). HRMS[ESI] calcd for C11H13N5F7 [M+H]^+^ 348.1054, found 348.1048.


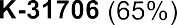


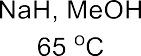
To a stirred solution of **2** (1.02 g, 4.0 mmol) in MeOH (10 mL) was added NaH (0.29 g, 4.4 mmol, 60% dispersed in mineral oil) at room temperature and the mixture was stirred for 30 min. Then, methyl chlorodifluoroacetate (0.44 mL, 4.2 mmol) was added to the reaction mixture. After stirring at 65 ^o^C for 18 h, the reaction was cooled to room temperature, quenched with sat. aqeous NH4Cl solution, and extracted with ethyl acetate (30 X 3 mL). The organic solution was dried over MgSO4, filtered, concentrated, and subjected to silica gel column chromatography (Hex:EtOAc = 5:1 to 1:1) to afford **K-31706** (0.82 g, 65%) as white solid.


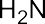

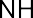

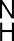

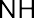

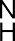

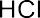

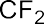

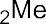

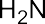

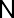

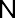

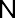

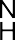

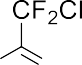


**6-(chlorodifluoromethyl)-N2-(4,4-difluorocyclohexyl)-1,3,5-triazine-2,4-diamine (K-31706)**; mixture of conformers (3:1); ^1^H NMR (600 MHz, CDCl3) δ 6.19 (br s, 3H), 5.91 (br s, 1H), 5.41-5.16 (m, 8H), 4.05 (m, 1H), 3.97-3.96 (m, 3H), 2.13-2.07 (m, 16H), 1.94-1.85 (m, 8H), 1.67-1.59 (m, 8H). ^13^C NMR (150 MHz, CDCl3) δ 168.5 (t, *J*C,F = 27.2 Hz), 168.0 (t, *J*C,F = 28.9 Hz), 167.4, 167.0, 166.0,

165.4, 122.5 (t, *J*C,F = 239.8 Hz), 122.4 (t, *J*C,F = 240.2 Hz), 121.9 (t, *J*C,F = 290.7 Hz), 121.8 (t, *J*C,F =

291.3 Hz), 47.7, 47.6, 32.11 (t, *J*C,F = 25.2 Hz), 32.07 (t, *J*C,F = 24.8 Hz), 28.7 (d, *J*C,F = 9.0 Hz), 28.3 (d,

*J*C,F = 9.2 Hz). HRMS[ESI] calcd for C10H13N5ClF4 [M+H]^+^ 314.0790, found 314.0799.

1. **^1^H-NMR, ^13^C-NMR, HRMS**


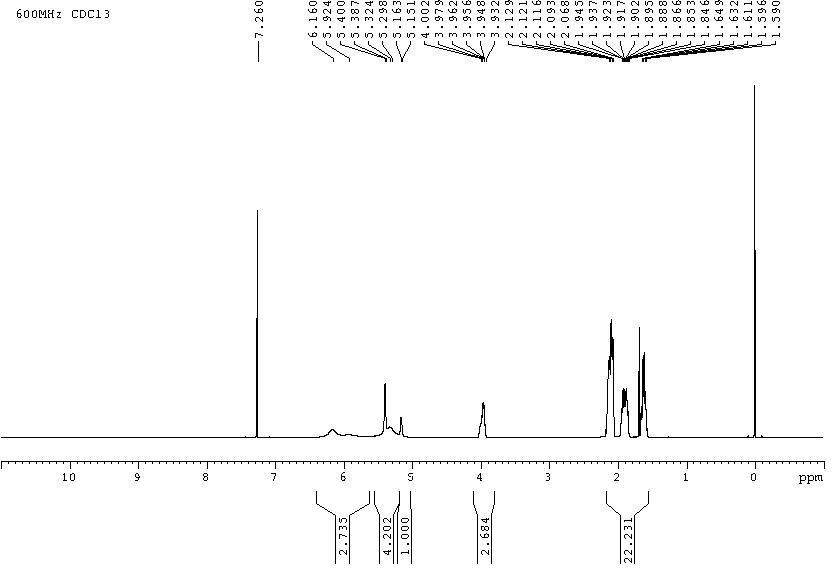

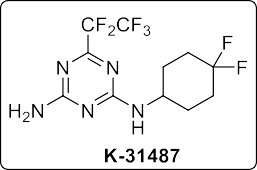

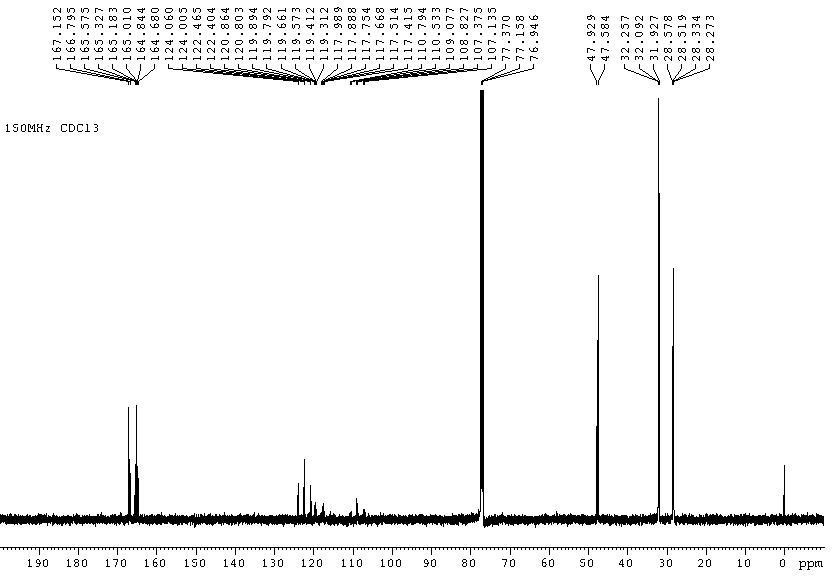

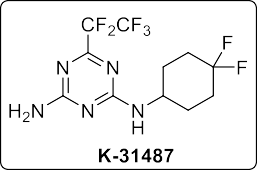

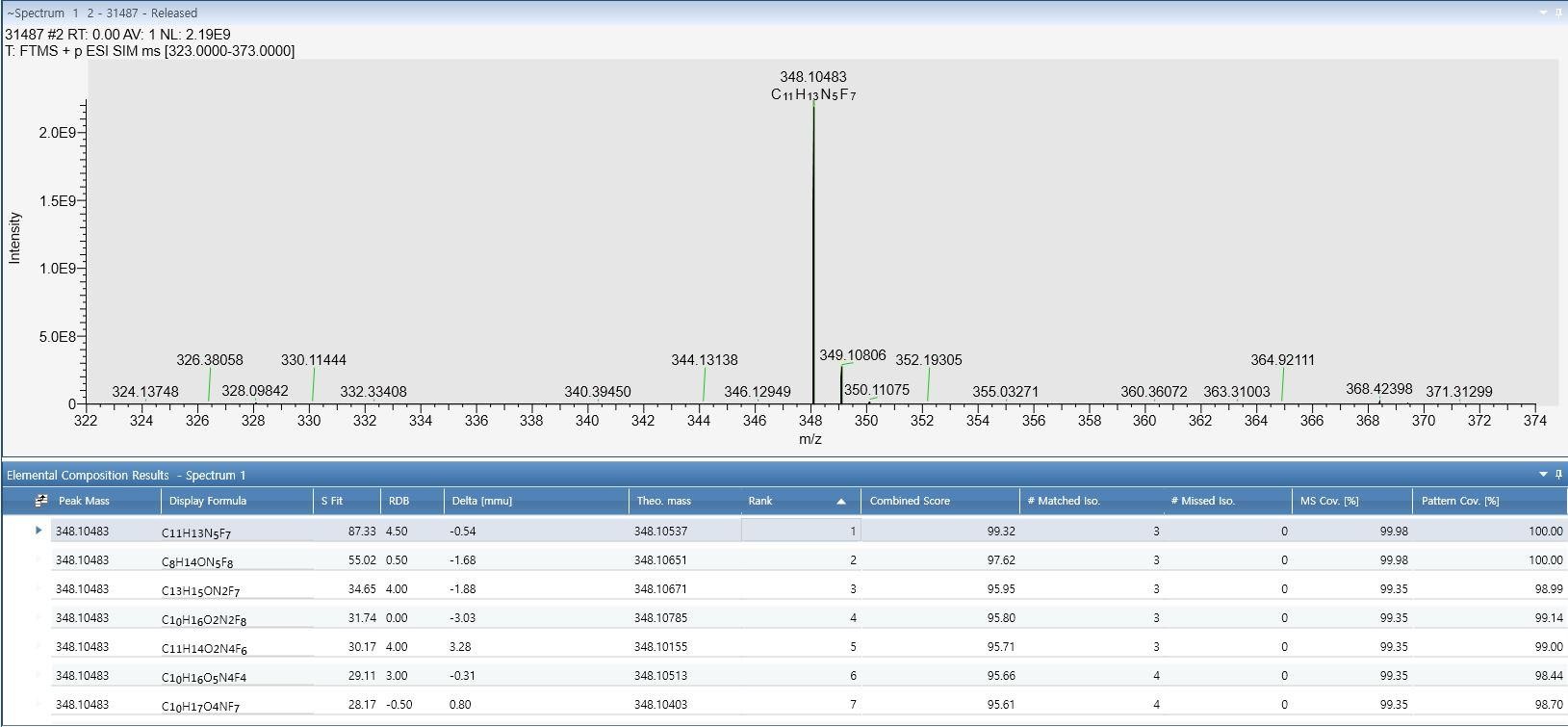

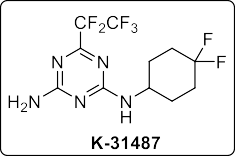

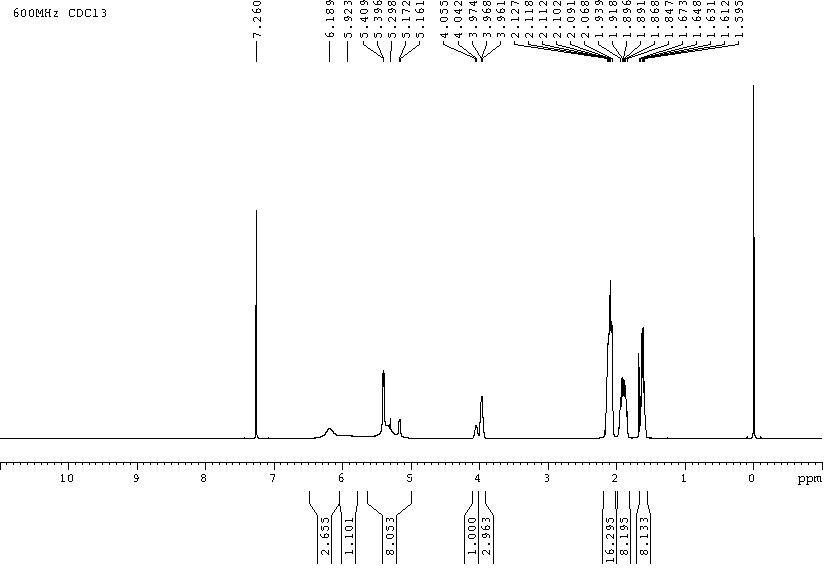

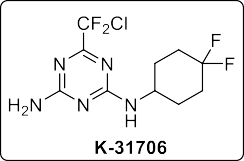

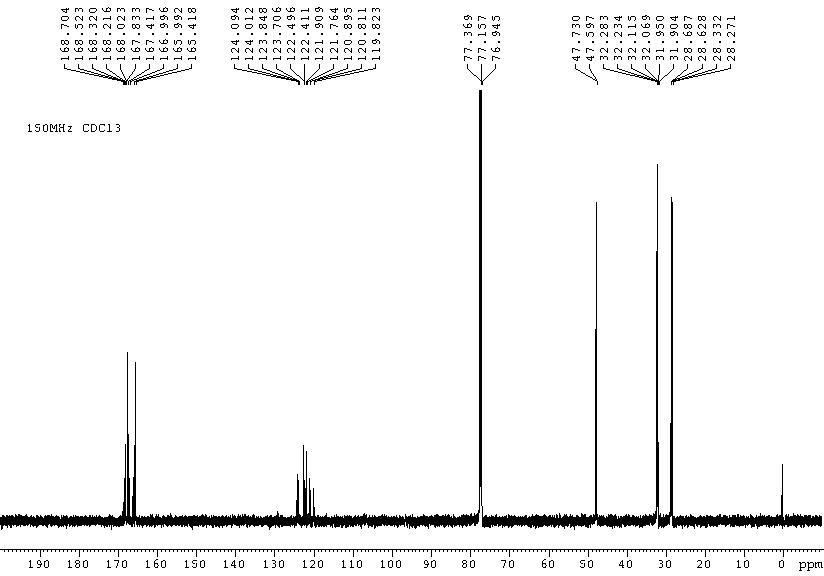

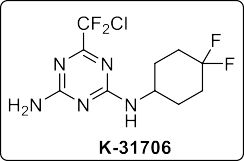

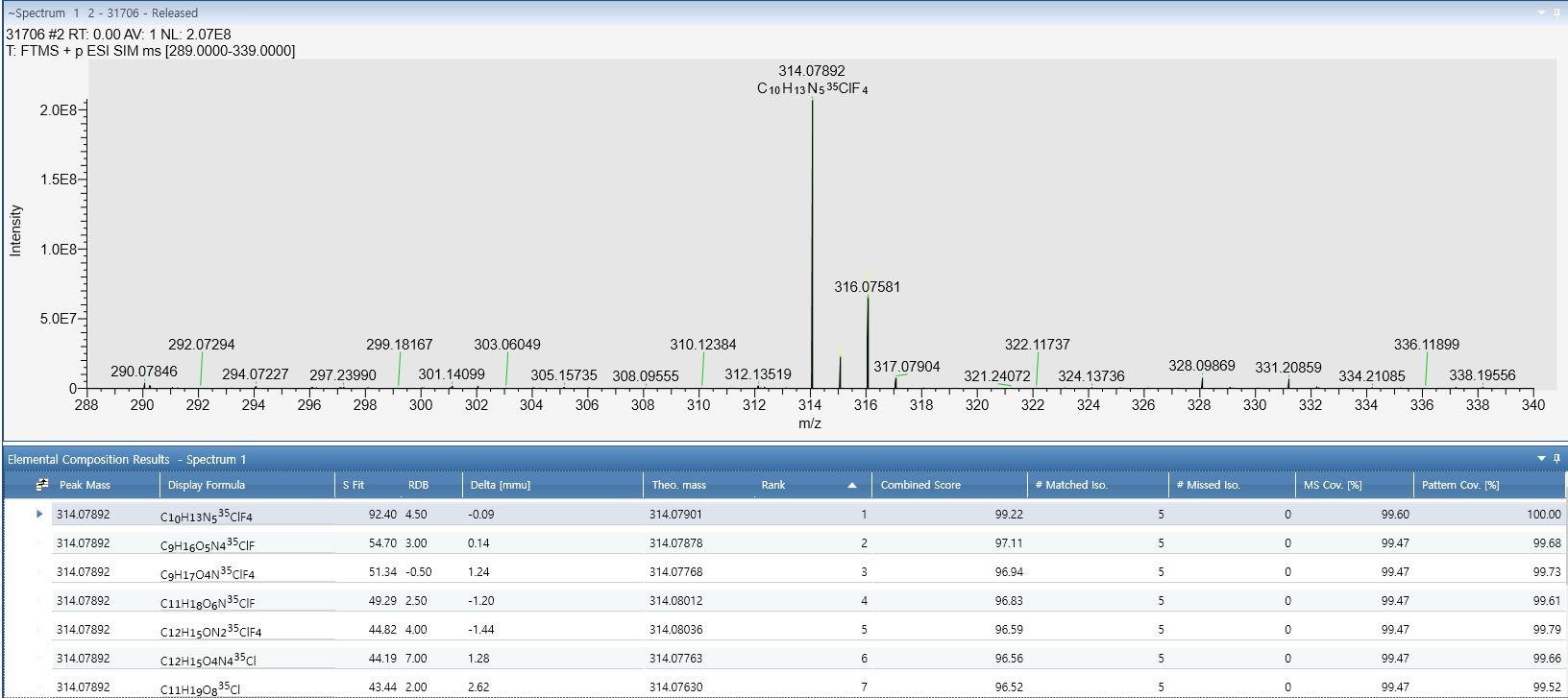

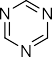

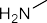

Supplement: Supplementary file 1 — Data S1: Supporting Information. [file PS-81-7219-s001.docx]
